# FWNNet: Presentation of a New Classifier of Brain Tumor Diagnosis Based on Fuzzy Logic and the Wavelet-Based Neural Network Using Machine-Learning Methods

**DOI:** 10.1155/2021/8542637

**Published:** 2021-11-22

**Authors:** Mohsen Ahmadi, Fatemeh Dashti Ahangar, Nikoo Astaraki, Mohammad Abbasi, Behzad Babaei

**Affiliations:** ^1^Department of Industrial Engineering, Urmia University of Technology, Urmia, Iran; ^2^Department of Electrical Engineering, Golestan University, Gorgan, Iran; ^3^Department of Computer Engineering, Shahid Beheshti University, Tehran, Iran; ^4^Department of Biomedical Engineering, School of Biological and Health Sciences, Arizona State University, Tempe, AZ, USA; ^5^School of Mechanical and Manufacturing Engineering, University of New South Wales, Sydney, NSW 2052, Australia

## Abstract

In this paper, we present a novel classifier based on fuzzy logic and wavelet transformation in the form of a neural network. This classifier includes a layer to predict the numerical feature corresponded to labels or classes. The presented classifier is implemented in brain tumor diagnosis. For feature extraction, a fractal model with four Gaussian functions is used. The classification is performed on 2000 MRI images. Regarding the results, the accuracy of the DT, KNN, LDA, NB, MLP, and SVM is 93.5%, 87.6%, 61.5%, 57.5%, 68.5%, and 43.6%, respectively. Based on the results, the presented FWNNet illustrates the highest accuracy of 100% with the fractal feature extraction method and brain tumor diagnosis based on MRI images. Based on the results, the best classifier for diagnosis of the brain tumor is FWNNet architecture. However, the second and third high-performance classifiers are the DT and KNN, respectively. Moreover, the presented FWNNet method is implemented for the segmentation of brain tumors. In this paper, we present a novel supervised segmentation method based on the FWNNet layer. In the training process, input images with a sweeping filter should be reshaped to vectors that correspond to reshaped ground truth images. In the training process, we performed a PSO algorithm to optimize the gradient descent algorithm. For this purpose, 80 MRI images are used to segment the brain tumor. Based on the results of the ROC curve, it can be estimated that the presented layer can segment the brain tumor with a high true-positive rate.

## 1. Introduction

A wavelet neural network (WNN) utilizes localized basis functions in the hidden layer to accomplish the required input-output mapping. The benefits of the WNN over the NN for the complex nonlinear system modeling are due to the integration of wavelet localization features with NN learning abilities [1]. The wavelet transform is capable of analyzing nonstationary data and revealing their local features. Neural networks can self-learn, which improves the model's accuracy. Fuzzy logic provides for the reduction of data complexity as well as the modeling of uncertainty and imprecision.

Nevertheless, type-1 fuzzy systems may be unable to manage rule uncertainty, in which case type-2 fuzzy systems may be used to solve the problem [1]. In WNNs, wavelet functions are utilized as activation functions in the hidden layer of the NN rather than local functions in time such as Gaussian and sigmoid functions. WNN structures are divided into two categories. Wavelets are used as activation functions in the initial one, derived from the continuous wavelet transform. As a result, the wavelet function's dilation and translation parameters can be any real-positive integer, and these parameters and the output layer weights can be changed. Wavelets as activation functions are derived from the discrete wavelet transform [2] of the second kind. FNNs are challenging to utilize to estimate unknown functions in the dynamics of hyperchaotic systems because they are high-dimensional nonlinear processes. The usage of wavelet neural networks is one of the easy ways to deal with these drawbacks (WNN). WNNs have been utilized in a variety of applications, including control [3], prediction [4, 5], forecasting [6, 7], and classification [8]. Due to its nonlinear structure and the presence of localized basis functions in the hidden layer of these neural networks, WNNs have been proven to operate well compared to traditional neural networks. TSK fuzzy models comprise a collection of rules, each serving as a “local model” by partitioning the input space into local fuzzy regions using fuzzy sets. These laws' consequences are expressed by a global function's constant or linear equation [9]. The wavelet concept is used in conjunction with fuzzy systems in many research studies. In literature, two forms of wavelet fuzzy neural networks have been suggested. The wavelet function is utilized as the activation function in the hidden layer of the neural network in the first one [10]. The wavelet function is employed in the subsequent section of TSK fuzzy systems in the second example. In these instances, the wavelet function replaces the linear function of the inputs [11, 12]. Various fuzzy neural network architectures are discussed, including radial basis function networks (RBFNs) [13] and multilayer perceptron (MLP). Fuzzy neural networks have recently been utilized for pattern recognition [14], image processing, approximations [15], identification [16], control [17], and other disciplines of science and industry. In addition, the fuzzy neural network's additional features such as time-series prediction [18], identification of nonlinear dynamical systems [12], dynamic fuzzy wavelet neural network [19], function learning [9], type-2 fuzzy wavelet neural network [20], indirect adaptive fuzzy wavelet neural network [21], and variable structure fuzzy wavelet neural network [22] are detailed in the following sections.

The objective of fuzzy models is to improve function estimate accuracy by lowering the number of wavelet components in the THEN section of fuzzy rules and the wavelet translation and dilation parameters. Furthermore, the possibility of using innovative methods of identification might alter precision. As a result, a new fuzzy wavelet neural network (FWNN) was developed. In certain studies, the gradient technique adapts and updates FWNN structural parameters such as the wavelet function's dilation and translation and network weights, resulting in greater competence and accuracy than WNNs. The use of adaptive learning algorithms based on the Lyapunov theory to update an adaptable FWNN structure [21, 23] has recently been proposed.

Consequently, the number of iterations required to train the FWNN structure is reduced, and function approximation precision is improved over NNs [24]. In this paper, the presented classifier is implemented in the brain tumor diagnosis. For feature extraction, a fractal model with four Gaussian functions is used.

## 2. Literature Review

In the presence of unknown dynamics, uncertainty in nonlinear system parameters, and external disturbances, Ebrahimi et al. developed a technique for creating a controller for nonlinear systems characterized by the Takagi–Sugeno (T-S) fuzzy model. The control law is divided into two sections. The first portion is based on the parallel distributed compensation (PDC) approach, which generates each control rule from the T-S fuzzy models relevant rule. The fuzzy wavelet neural network (FWNN) estimator, which is triggered by the premise of wavelet transformation multiresolution analysis (MRA) and fuzzy notions, is found in the second section. In the T-S fuzzy model, it is accurate to predict uncertainties and external disturbances. The T-S fuzzy models suggested approach for observer-based controller design for uncertain nonlinear systems has been improved [24]. Zirkohi and Shoja-Majidabad [25] suggested an efficient adaptive control technique to explore synchronizing two chaotic systems. This method resulted in a type-2 fuzzy wavelet neural network that can better estimate unknown variables and external disturbances in chaotic system dynamics. An optimum robust control term was also introduced to the suggested controller to increase its resilience against unknown system disturbances and uncertainties. As a model-free controller, this method has many advantages. It was determined that the suggested control approach could ensure the synchronization and stability of the closed-loop control system [25], employing the Lyapunov stability theory and transient performance analysis.

Peker [26] created a deep learning-based hybrid model for hyperspectral image categorization. A convolutional neural network (CNN) was utilized to derive multilayer picture representation. A complex-valued wavelet neural network (CVWNN) was used to categorize the image by utilizing the recovered features. The process steps of the recommended technique are listed below. First and foremost, hyperspectral pictures were subjected to the CNN algorithm. This stage has resulted in the acquisition of efficient characteristics. A unique random-based transformation approach was used to convert the collected characteristics into a complex-valued number format. As a result, a new complex-valued attribute set for the HSI classification has been discovered. The CVWNN algorithm has been given the acquired features as input. For improving CNN's resilience and generalization, the hybrid approach substitutes the real-valued neural network with the CVWNN. Huang et al. created a hybrid fuzzy wavelet neural network (FIWN) by combining polynomial neural networks (PNNs) with fuzzy inference-based wavelet neurons (HFWNN). The fuzzy set inference-based wavelet neurons (FSIWNs) and fuzzy relation inference-based wavelet neurons (FRIWNs) are two forms of FIWNs that have been suggested. A wavelet neuron (WN) is a FIWN lacking any fuzzy set components (e.g., the hypothesis portion of a fuzzy rule). The variables of wavelet functions in FIWNs or WNs have started utilizing the C-means clustering approach to overcome the constraints of traditional wavelet neural networks or fuzzy wavelet neural networks whose variables are generated on a purely random basis. The following are the key tactics used in the development of HFWNN. FIWNs (for instance, FSIWN or FRIWN) make up the network's initial layer, which indicates data uncertainty. The second and higher layers are composed of WNs, which are flexible and can accomplish a linear combination of wavelet functions. Second, genetic optimization is utilized to fine-tune the parameters employed in the HFWNN's design [27]. According to Golestaneh et al., each fuzzy rule relates to a subwavelet neural network composed of wavelets with varying dilations and translations. In the THEN part of each fuzzy rule, one coefficient is assessed per every two inputs to achieve a compromise between network complexity and performance accuracy. This article first establishes the equality of an FW model and an SLFN, following which ELM may be applied directly to the model. All wavelet coefficients and free membership function parameters are generated at random. Using a one-pass learning approach, only the output weights are calculated analytically. On different benchmark datasets, FW-ELM is compared to prominent fuzzy models such as OS-Fuzzy-ELM, Simple TS, ANFIS, and numerous other significant algorithms such as ELM, BP, and SVR.  Table 1 shows the summary of some utility of the fuzzy wavelet neural network [28].

## 3. Methods and Materials

**Figure 1:**
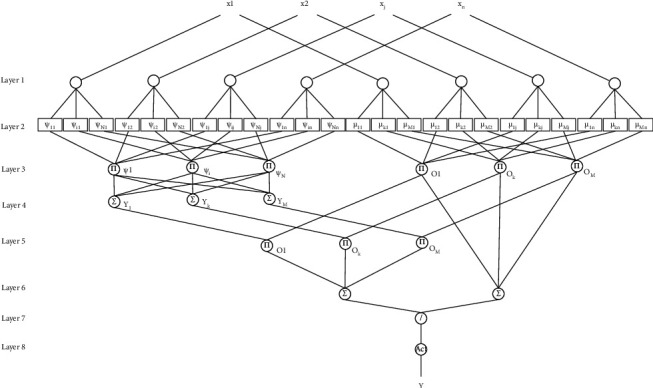
The FWNNet architecture for feature categorization.

**Figure 2:**
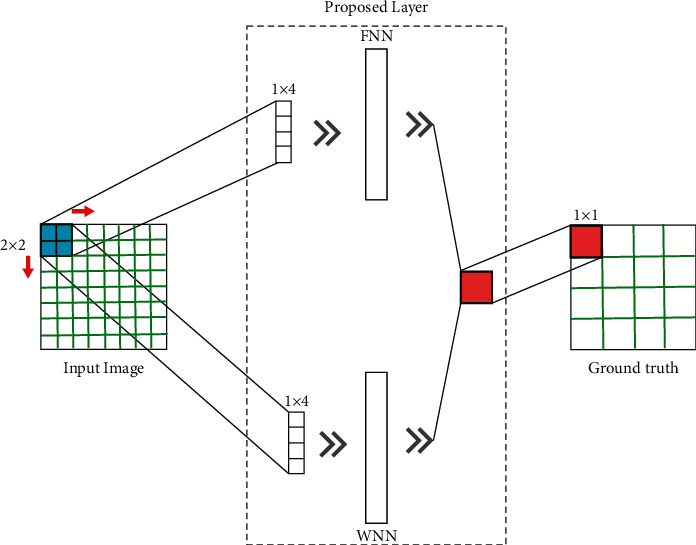
The FWNNet layer's architecture in deep learning.

For classification purposes, the suggested network combines a fuzzy neural network and a wavelet neural network. The network has eight levels, according to the suggested method's design (see Figures 1 and 2).

(1) First Layer. The input characteristics of the issue are the first layer of the proposed FWNNet. Independent variables, picture classification features, and time series are all included. *X*={*X*_*j*_*|j*=1,…, *n*} can be used to display it.
(2) Second Layer. The second layer comprises wavelet neural networks and fuzzy neural networks for an estimate. The wavelet is computed using the equation in the wavelet portions of the layer:
  (1) $$\begin{array}{c} \psi_{ij}^{k}=\psi \left(\frac{x_{j}-b_{ij}^{k}}{a_{ij}^{k}}\right),\;i=1,\ldots ,N,\;j=1,\ldots ,n. \end{array}$$
The number of wavelets is *N*, while the number of input features is *n*. Wavelet transformations can simultaneously display functions and disclose their local characteristics in the time-frequency domain. These characteristics make it easier to train neural networks to model extremely nonlinear data accurately. This is how the wavelet is written:
  (2) $$\begin{array}{c} \psi_{a,b}={\left|a\right|}^{-\left(1/2\right)}\psi \left(\frac{x-b}{a}\right),\;a,b\in R,\;a\neq 0, \end{array}$$
where *ψ*(*x*) ∈ *L*^2^(*R*) is the wavelet function depending on the equation
  (3) $$\begin{array}{c} C_{\psi }=\int_{0}^{+\infty }\frac{\left|\hat{\psi }\left(\omega \right)\right|}{\omega }\text{d}\omega <+\infty . \end{array}$$
Assume $\hat{\psi }\left(\omega \right)$ is the Fourier transform of *ψ*(*x*). To mimic multivariable processes, multidimensional wavelets must be developed.
Furthermore, the fuzzy membership function is computed in the fuzzy regions of the second layer using the following equation:
  (4) $$\begin{array}{c} \mu_{kj}=e^{-{\left(\left(X_{j}-c_{kj}\right)/\sigma_{kj}\right)}^{2}}, \end{array}$$
where *c*_*kj*_ denotes the centers and *σ*_*kj*_ is the standard deviation for the rule *k* membership function.
(3) Third Layer. The outputs of layer 3 must be multiplied together in the third layer, the aggregation layer. In layer 3 of the rules, multiple WNNs with *N*_*k*_ wavelet activation functions are employed in the wavelet portions:
  (5) $$\begin{array}{c} \Psi_{i}^{k}=\prod_{j=1}^{n}\psi_{ij}^{k},\;k,1,\ldots ,M. \end{array}$$
In addition, each node in layer 3 indicates one fuzzy rule. The output signals (6) are calculated using the AND operator:
  (6) $$\begin{array}{c} O_{k}=\prod_{j=1}^{n}\mu_{kj},\;k,1,\ldots ,M. \end{array}$$
(4) Fourth Layer. The result of the wavelet components is computed at the fourth layer. The following is the overall structure of the rules:
  (7) $$\begin{array}{c} R_{k}:\text{IF }x_{1}\text{ is }A_{k1}\ldots \text{AND }x_{n}\text{ is }A_{kn},\\ \text{THEN}\;Y_{k}=\sum_{i=1}^{N_{k}}w_{i}^{k}\Psi_{i}^{k}+{\bar{y}}_{k}. \end{array}$$
Let *x*_1_, *x*_2_,…, *x*_*n*_ represent the input feature, *Y*_1_, *Y*_2_,…, *Y*_*M*_ represent the fourth layer output layer, and *A*_*kj*_ represents the *k*th fuzzy set with normal membership. The matrix of weights and the bias are stored in this hidden layer as *w*_*i*_^*k*^ and ${\bar{y}}_{k}$.
(5) Fifth Layer. The outputs of the fuzzy neural network in the third layer *O*_*k*_ and the result of the fourth layer of the wavelet neural network *Y*_*k*_ are combined in the fifth layer. The defuzzification inference is discussed in layers 5–8. Layer 5 multiplies layer 3's output data by layer 4's output data.
(6) Sixth Layer. Two neurons act as summing operators for layer 5 and layer 3 output signals, respectively, at this layer. The quotient is generated by layer 7's output neuron, which shows each wavelet neural network's output proportion to the proposed-ultimate FWNNet's output.
  (8) $$\begin{array}{c} O_{k}^{\left(5\right)}=O_{k}^{\left(3\right)}\cdot O_{k}^{\left(4\right)}=O_{k}\cdot Y_{k},\\ O_{1}^{\left(6\right)}=\sum_{k=1}^{M}O_{k}^{\left(5\right)},\\ O_{2}^{\left(6\right)}=\sum_{k=1}^{M}O_{k}^{\left(3\right)}. \end{array}$$
(7) Seventh Layer. The output of the outputs is collected at the seventh layer.
  (9) $$\begin{array}{c} y=O^{\left(7\right)}=\frac{O_{1}^{\left(6\right)}}{O_{2}^{\left(6\right)}}=\frac{\sum_{k=1}^{M}O_{k}Y_{k}}{\sum_{k=1}^{M}O_{k}}. \end{array}$$
(8) Eighth Layer. This is the network's final layer for the categorization of the feature. It is an activation function that converts data into output layer values. The round function is provided in our suggested model. The gradient descent approach is utilized for training the proposed-FWNNet after variable calibration utilizing inline-PSO. The gradient of the objective variable is calculated in the opposite direction based on $\Theta =\left(c_{kj},\sigma_{kj},b_{ij}^{k},a_{ij}^{k},w_{i}^{k},{\bar{y}}_{k}\right)$ as follows:
  (10) $$\begin{array}{c} E\left(\Theta ,x,y\right)=\frac{1}{2}{\left(y-f\right)}^{2},\\ \Theta \left(t+1\right)=\Theta \left(t\right)+\Delta \Theta ,\\ \Delta \Theta =\left(-\gamma_{c}\frac{\partial E}{\partial c_{kj}},-\gamma_{\sigma }\frac{\partial E}{\partial \sigma_{kj}},-\gamma_{b}\frac{\partial E}{\partial b_{ij}^{k}},-\gamma_{a}\frac{\partial E}{\partial a_{ij}^{k}},-\gamma_{w}\frac{\partial E}{\partial w_{i}^{k}},-\gamma_{\bar{y}}\frac{\partial E}{\partial {\bar{y}}_{k}}\right). \end{array}$$

## 4. Results and Discussion

### 4.1. Data Collection

A brain tumor is one of the most dangerous illnesses that may affect both children and adults. Benign tumors, malignant tumors, pituitary tumors, and other types of brain tumors are categorized. Proper therapy, planning, and precise diagnostics should be performed to increase the patients' life expectancy. MRI is the most effective method for detecting brain cancers. During the scans, an enormous amount of picture data is created. A radiologist examines these pictures. Because of the complexity of brain tumors and their characteristics, a manual examination might be subject to mistakes. This study utilized the Brain Tumor Classification (MRI) dataset from Kaggle to identify and detect brain tumors [34]. Furthermore, we applied the suggested method to five different brain diseases. Alzheimer's, Glioma, Huntington's, Meningioma, and Sarcoma are among the diseases included in the database. MRI images from Harvard Medical School's repository [35] are examples of illness imaging. All images are 256 × 256 pixels and are from T2-weighted MR brain imaging in the axial plane. Each image is analyzed and processed independently using an unsupervised method.

### 4.2. Feature Extraction

**Figure 3:**
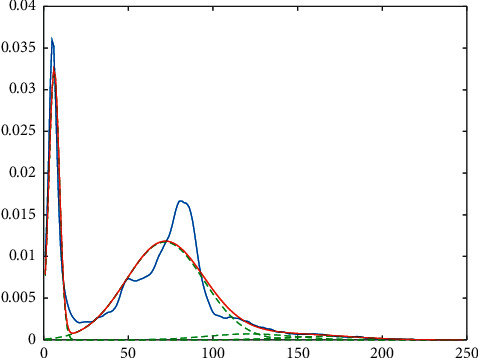
Modeling of an image using fractal feature extraction method.

The fractal method was used with covariance analysis to generate eigenvalues from the picture and reduce the dimension. One picture is a two-dimensional matrix and a single vector in the fractal method, which needs identical input images. Grayscale images with a certain resolution are required. By reshaping matrices, each image is transformed into a column vector. An *M* × *N* matrix was used to take the images. The number of images is *M*, and every image has a pixel value of *N*. To determine the normal distribution of each original image, the average image must be determined. The covariance matrix may then be calculated, and the covariance matrix's eigenvalues and eigenvector can be created. In the fractal system, *M* denotes the number of training pictures, *F*_*i*_ is the average of the images, and *l*_*i*_ denotes each image in *T*_*i*_. In the beginning, there are *M* pictures, each of which is *N* × *N* pixels in size [36, 37]. We used a summation of four Gaussian functions to model image histograms using the fractal feature extraction approach. As a result, each image has four characteristics.  Figure 3 shows an instance of a feature extraction histogram.

According to Figure 3, the blue line is the input image's histogram, and the total of the four green Gaussian functions should match the blue line. The red line represents the summing of the normal functions.

### 4.3. Classification Results

**Figure 4:**
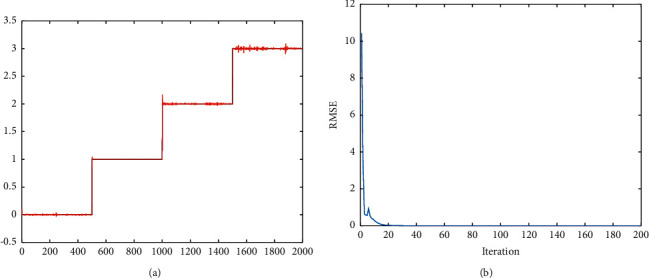
RMSE value in the training process is based on numerical labels. Findings of classification utilizing presented FWNNet: (a) output labels over modeled labels and (b) RMSE value in the training process based on numerical labels.

**Figure 5:**
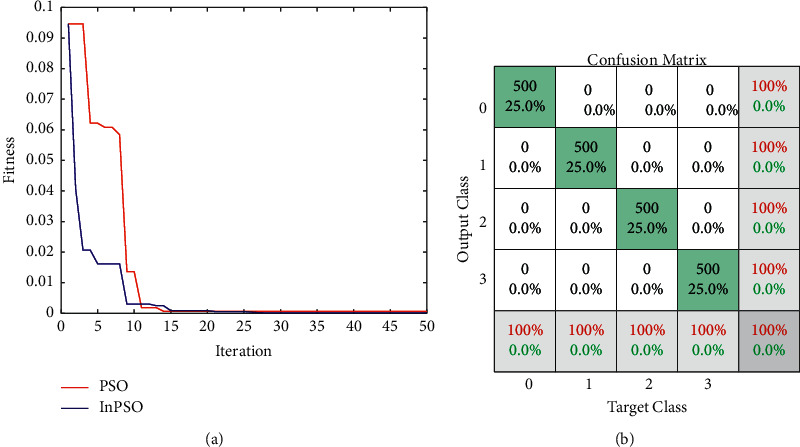
The findings of classification using the PSO and InPSO optimized methods: (a) fitness value and (b) confusion matrix.

In this section, we used the presented FWNNet method for the classification of brain tumors. The suggested FWNNet layer architecture is shown in Figure 2. The dataset includes four classes of images including (0) Normal tissue, (1) Glioma, (2) Meningioma, and (3) Pituitary Tumor. In this paper, we used the presented FWNNet to classify the tumors. The results of classification are presented in Figures 4 and 5.

The output labels 0, 1, 2, and 3 are simulated by utilizing input fractal features in the FWNNet technique findings in Figure 4. We used 2000 PNG pictures of the brain MRI to train the model. According to the findings, the provided approach can accurately predict the output value. In the following portion of the hybrid learning scheme, the RMSE reduction curve during training and testing of the gradient descent method is shown in Figure 4(b). In addition, Figure 4 depicts the test signal's actual and anticipated outputs (Figure 4(a)). As demonstrated in Figure 4, when iteration modest learning rates are used, the RMSE values can drop gradually with iteration owing to proper initialization of the network in the stage of two-layer inline-PSO, whose adjustment method is coordinated with the subsequent gradient descent.

Each WNN in our research has two rules (*M* = 2) and two wavelet neurons (*N*_*k*_ = 2; *k* = 1; 2). The number of variables that may be changed is *N* = 30. The FWNNet is trained using the hybrid learning method. The optimization results of inline-PSO and basic PSO are compared and illustrated in Figure 5(a). To save time and avoid overtraining the training signal, the population size psize = 20, and the termination iteration number Maxgen = 50 is kept low. This might cause a restricted testing signal search region. The linear decreasing inertia weight is utilized, with both c1 and c2 acceleration coefficients set to 2. Inline-PSO has a slower convergence rate than PSO, as illustrated in Figure 5(a).

**Figure 6:**
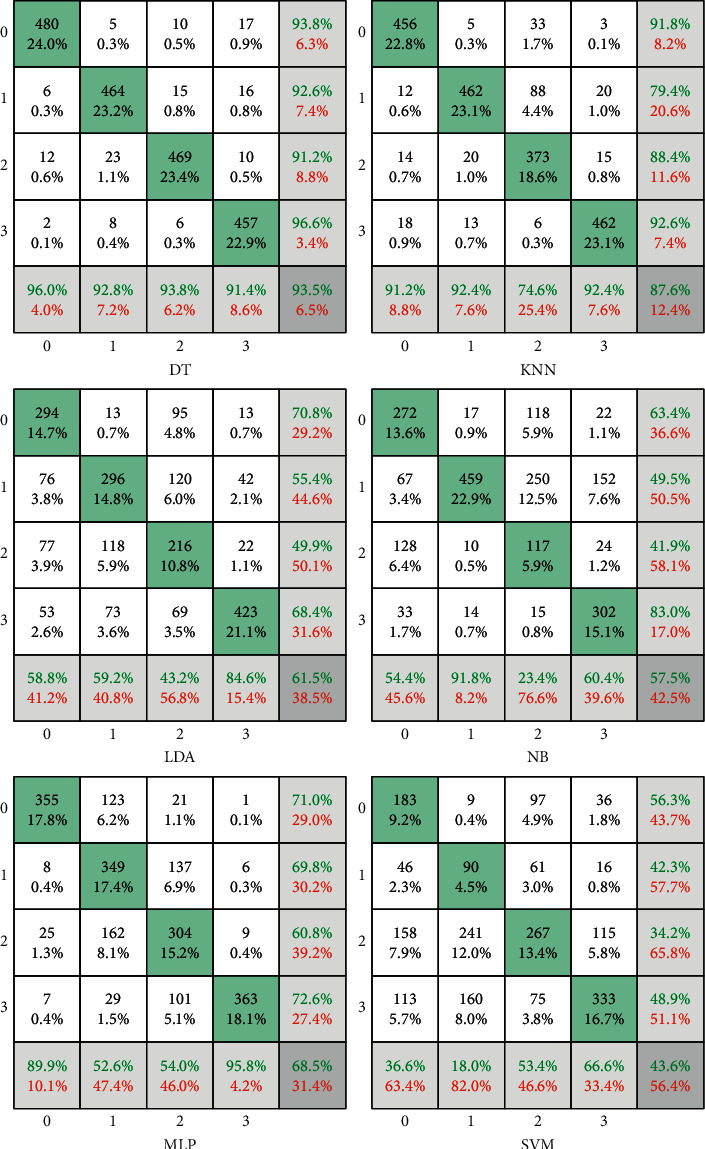
The results of the machine learning classifiers based on fractal feature extraction.

The confusion matrix of the classification based on the presented FWNNet is presented in Figure 5(b). In the confusion matrix, the accuracy of the proposed method for diagnosing the brain tumor is 100%. It means that all images are detected with high sensitivity and precision. Based on the fractal feature extraction method, the proposed classifier is compared with other machine learning classifiers including decision tree (DT), K-nearest neighbor (KNN), linear discrimination analysis (LDA), naïve Bayes (NB), multilayer perceptron (MLP), and support vector machine (SVM). The results of the traditional classifiers are illustrated in Figure 6.

In Figure 6, the green cells show the true value of the classification, and white cells represent the false results. Moreover, horizontal gray cells illustrate the sensitivity value of the diagnosis of each class, and the vertical gray cells are the precision values. Finally, the corner cell shows the accuracy value of the classifiers. Based on the outcome of the DT classifier, from 500 normal brain tumors, 480 (96%) images are detected correctly. Thus, 6 of them were classified in Glioma, 12 in Meningioma, and 2 in Pituitary class. Based on this classifier, the sensitivity of the DT for detecting the Normal tissue, Glioma, Meningioma, and Pituitary Tumor is 96%, 92.8%, 93.8%, and 91.4%, respectively. To compare with other classifiers, the DT results in the highest accuracy of 93.5%. However, the KNN methods show the second highest accuracy of 87.6%. In this classifier, the lowest sensitivity is found in the Meningioma tumor. Regarding this result, from 500 images of Meningioma tumor, 373 of them were detected successfully. Also, the lowest precision belongs to the meningioma with an 88.4% value. This value means that from all images detected as Meningioma, 373 of them are Meningioma. However, 15, 20, and 14 are long for Pituitary, Glioma, and Normal tissue. Compared with other machine learning classifiers, the lowest accuracy has resulted in the SVM approach with 43.6%. Regarding the results, the accuracy of the DT, KNN, LDA, NB, MLP, and SVM is 93.5%, 87.6%, 61.5%, 57.5%, 68.5%, and 43.6%, respectively. Based on the results, the presented FWNNet illustrates the highest accuracy of 100% with the fractal feature extraction method and brain tumor diagnosis based on MRI images. For the best illustration of this comparison, the ROC curve (receiver operating characteristic curve) shows the performance of each classifier. This curve is plotted based on the true-positive rate versus the false-positive rate. The best classifier has the highest true-positive rate and lowest false-positive values based on this curve.

**Figure 7:**
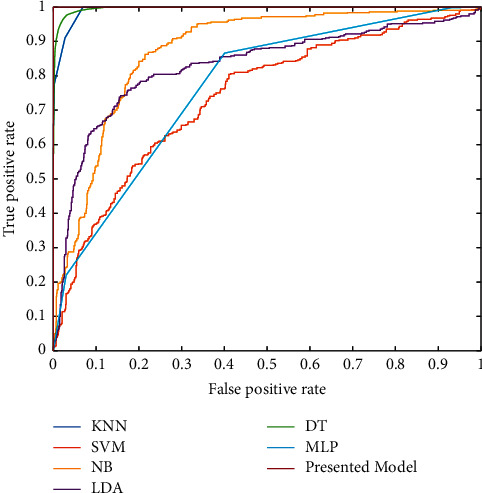
The ROC curve of the classifiers for diagnosis of the brain tumor.

The ROC curve is illustrated in Figure 7. Based on the results, the best classifier for the diagnosis of brain tumor is the presented FWNNet architecture. However, the second and third high-performance classifiers are the DT and KNN, respectively.

### 4.4. Segmentation Results

**Figure 8:**
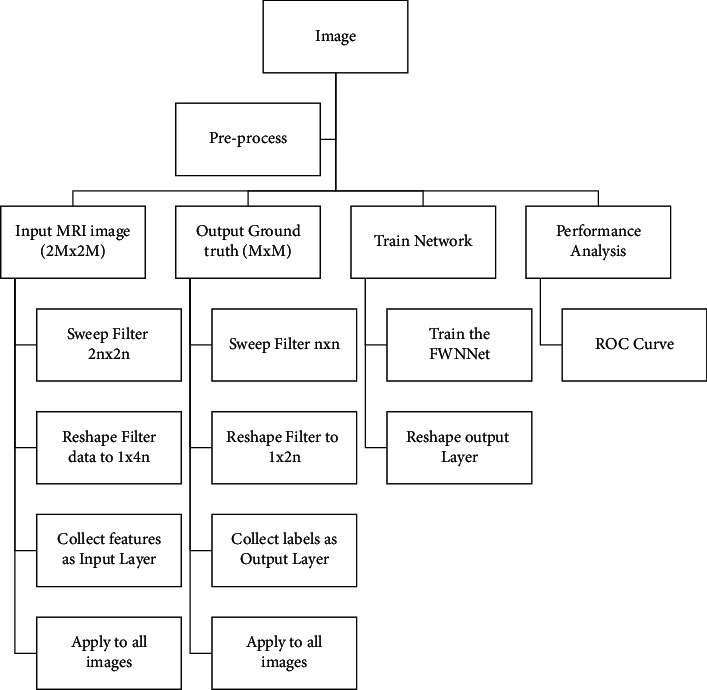
The flowchart of the presented method for the supervised segmentation method.

In this part of the paper, we developed the FWNNet architecture for the segmentation of brain tumors.  Figure 8 shows the flowchart of the presented method for the supervised segmentation method. Based on the architecture represented in Figure 2, the segmentation method has the following steps. First, we need two images, including input images with RGB and grayscale with a two-dimensional scale and a ground truth image. In the ground truth image, the tumor place should be labeled with the pixel value of 255. Based on the intrinsic structure of fuzzy logic and wavelet transformation, it is better to transform an image into an image that lowers the number of zeros.

**Figure 9:**
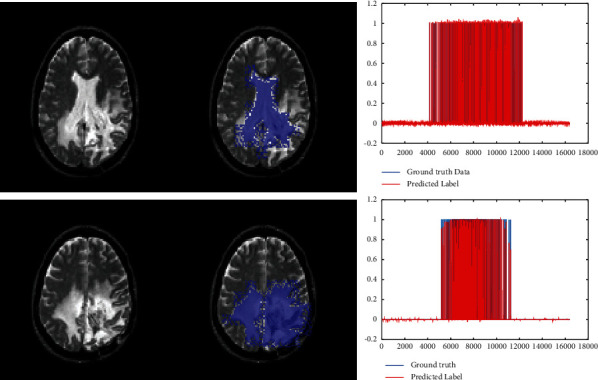
The results of segmentation based on the presented FWNNet layer.

Therefore, in this paper, the input images are transformed with the Gabor filter. Based on the architecture of the FWNNet layer, the size of the input image is shown to be higher than ground truth images so that the input matrix with 2*M* × 2*M* size should have corresponded to the ground truth image with the size of *M* × *M*. In the training process, input images with a sweeping filter should be reshaped to vectors that correspond to reshaped ground truth images. In the training process, we performed a PSO algorithm to optimize the gradient descent algorithm. Finally, the ROC curve illustrates the performance of the presented segmentation methods. The results of segmentation are represented in Figure 9. In this figure, the left images are the input images that are segmented using the presented FWNNet layer.

**Figure 10:**
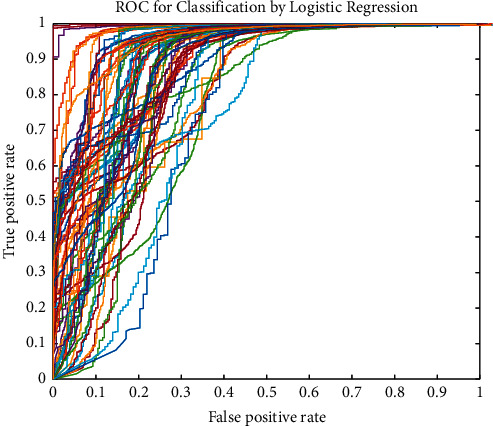
The ROC curve of the presented supervised segmentation method.

The results of the segmented tumor are illustrated in the middle column of Figure 9. The ground truth image in the vector is illustrated in the right column of Figure 9. Based on the results of the presented method, the predicted ground truth values are almost equal to target values. In this paper, 80 images are used to segment the brain tumor. The performance of the presented method for each image is depicted in Figure 10. Based on the results of the ROC curve, it can be estimated that the presented layer can segment the brain tumor with a high true-positive rate.

## 5. Conclusion

A new classifier dependent on fuzzy logic and wavelet transformation in a neural network was described in this study. A layer in this classifier predicts the numerical characteristic associated with labels or classifications. The proposed classifier is used to diagnose brain tumors. A fractal model with four Gaussian functions is utilized to extract features. A total of 2000 MRI pictures are used in the categorization. According to the results of the DT classifier, 480 (96 percent) pictures from 500 typical brain tumors are accurately recognized. As a result, six were categorized as Glioma, twelve as Meningioma, and two as Pituitary. According to this classifier, the DT's sensitivity for recognizing Normal tissue, Glioma, Meningioma, and Pituitary Tumor is 96 percent, 92.8 percent, 93.8 percent, and 91.4 percent, respectively. When compared to other classifiers, the DT has the greatest accuracy (93.5%). The KNN techniques, on the other hand, have the second greatest accuracy of 87.6 percent. The Meningioma tumor has a minor sensitivity in this classifier.

In this case, 373 images of Meningioma tumors were effectively identified out of 500 total images. Meningioma also has the lowest precision, with an 88.4 percent value. This result indicates that 373 of the pictures identified as Meningioma are Meningioma. 15, 20, and 14 of them, on the other hand, are looking for Pituitary, Glioma, and Normal tissue, respectively. The SVM method has the least accuracy of 43.6 percent when compared to other machine learning classifiers. The accuracy of the DT, KNN, LDA, NB, MLP, and SVM, respectively, is 93.5 percent, 87.6 percent, 61.5 percent, 57.5 percent, 68.5 percent, and 43.6 percent. According to the findings, the given FWNNet demonstrates the maximum accuracy of 100 percent using the fractal feature extraction approach and brain tumor identification based on MRI scans. According to the findings, the given FWNNet demonstrates the maximum accuracy of 100 percent using the fractal feature extraction approach and brain tumor identification based on MRI scans. According to the findings, the FWNNet architecture is offered as the best classifier for brain tumor diagnosis. The DT and KNN, on the other hand, are the second and third high-performance classifiers, respectively. For the segmentation of brain tumors, the described FWNNet technique is used. We offer a unique supervised segmentation approach depending on the FWNNet layer in this work. First, we will need RGB and grayscale input images with two-dimensional scales and a ground truth image. The input pictures are converted with a Gabor filter in this article. Sweeping filters must reshape input pictures into vectors that match altered ground truth images during the training phase. We used a PSO method to optimize the gradient descent technique throughout the training procedure. Ultimately, the ROC curve depicts the performance of the segmentation methods provided. The brain tumor is segmented using 80 MRI scans for this reason. The provided layer may segment the brain tumor with a high true-positive rate, according to the findings of the ROC curve.

## Figures and Tables

**Table 1:** Summary of some utility of fuzzy wavelet neural network.

Author	Year	Method	Goal	Utility	Results
Ghoushchi et al. [29]	2021	Fuzzy wavelet neural network	Forecasting	Forecasting of short-term wind power	The findings revealed that the suggested technique was a more efficient tool with greater precision for short-term wind power forecasting than previously published methods.
Shao et al. [30]	2021	Fuzzy wavelet neural control	Control	Control of micro-electro-mechanical system gyroscope	The effectiveness of the control technique was confirmed by simulation findings and comparisons
Hamedani et al. [31]	2021	Recurrent fuzzy wavelet neural network	Control	Control of robotic manipulators	In the case of significant disturbances, the suggested fuzzy gain dynamic surface was utilized to force the manipulator's end-effector to track the required impedance profile
Ebrahimi et al. [24]	2021	Fuzzy wavelet neural network	Control	Observer-based controller design for uncertain nonlinear systems	Without using the usual conservative lemma or considering constraints on uncertainties, the suggested controller managed the uncertainties and external disturbances in the T-S fuzzy model
Luo et al. [32]	2021	Fuzzy wavelet neural network	Dynamical analysis	Self-sustained electromechanical seismograph system	The suggested scheme's efficacy and benefits were demonstrated through numerical simulation
Abiyev and Abizada [33]	2021	Type-2 fuzzy wavelet neural network	Prediction	Energy performance of residential buildings	The obtained findings suggested that the T2FWNN system may be used to estimate energy performance and anticipate energy consumption in residential structures
Zirkohi and Shoja-Majidabad [25]	2021	Type-2 fuzzy wavelet neural network	Dynamical analysis	Estimating the unknown terms and the external disturbance in the chaotic systems' dynamics	The suggested technique outperforms radial basis function neural networks in simulations, demonstrating its advantages in secure communication applications
Peker [26]	2021	Fully complex-valued wavelet neural network	Classification	Classification of hyperspectral imagery	Three data sets containing three popular hyperspectral aerial pictures were used in the tests. When compared to previous classification methods, the proposed method improved classification accuracy
Huang et al. [27]	2018	Hybrid fuzzy wavelet neural networks	Prediction	Fuzzy inference-based wavelet neurons	When compared to the outcomes provided by several prior well-known and widely utilized neurofuzzy models, experimental experiments including three extensively used data sets reveal some encouraging findings
Golestaneh et al. [28]	2018	Fuzzy wavelet extreme learning machine	Prediction, classification, and dynamic analysis	Base method	While the number of linear learning parameters is reduced and SDs are lower, the performance of FW-ELM is equivalent to that of OS-fuzzy-ELM and better than other published works for classification and regression tasks

## Data Availability

The MRI data used in this study are the BraTS dataset (https://www.kaggle.com/).
